# Assessment of Co-Formulants in Marketed Plant Protection Products by LC-Q-Orbitrap-MS: Application of a Hybrid Data Treatment Strategy Combining Suspect Screening and Unknown Analysis

**DOI:** 10.1021/acs.jafc.2c01152

**Published:** 2022-06-07

**Authors:** Antonio Jesús Maldonado-Reina, Rosalía López-Ruiz, Roberto Romero-González, José Luis Martínez Vidal, Antonia Garrido-Frenich

**Affiliations:** Research group “Analytical Chemistry of Contaminants”, Department of Chemistry and Physics, Research Centre for Mediterranean Intensive Agrosystems and Agri-Food Biotechnology (CIAMBITAL), University of Almería, Agri-Food Campus of International Excellence, ceiA3, 04120 Almería, Spain

**Keywords:** pesticide formulations, characterization, additives, UHPLC-HRMAS, nontargeted analysis

## Abstract

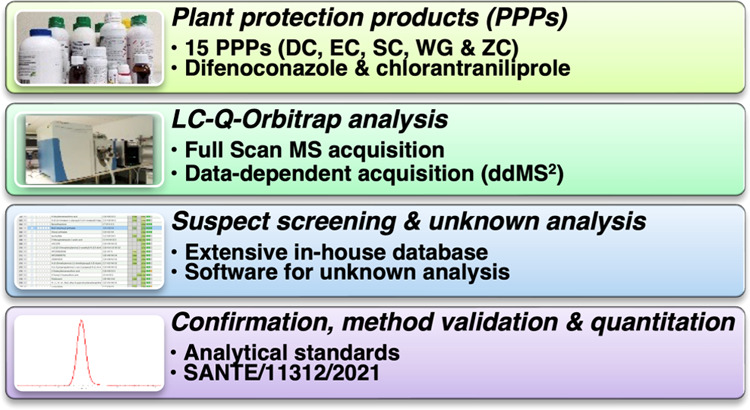

The aim of this study was the determination of co-formulants in 15 different chlorantraniliprole- and difenoconazole-based plant protection products (PPPs) belonging to different formulations. Samples were analyzed by ultrahigh-performance liquid chromatography coupled to Q-Orbitrap high-resolution mass accuracy spectrometry (UHPLC-Q-Orbitrap-MS), operating in full-scan MS and data-dependent acquisition (ddMS^2^) modes. A total of 78 co-formulants were tentatively identified by a combination of suspect screening and unknown analysis. Nine of them were later confirmed by analytical standards. Finally, the analytical method was successfully validated and co-formulants were quantified. Linear alkyl ethoxylates (LAS) were the most common type of co-formulant, followed by sodium alkylbenzene sulfonates. Moreover, sodium dodecyl benzene sulfonate had the highest concentration of any co-formulant (up to 32.33 g/L). In all, an innovative identification of co-formulants in a large number of PPPs is presented, which will give room for future studies delving into the composition of PPPs or determining these co-formulants in environmental or agricultural samples.

## Introduction

Plant protection products (PPPs) have long been an essential resource for effective pest control. It is estimated that the application of pesticides prevents the loss of 78% of fruit crops, 54% of vegetable crops, and 32% of cereal crops, which would have a devastating impact on human nutrition and economy.^1^ According to the most recent EUROSTAT pesticide sales data, as many as 352 tons of PPPs were sold in EU-27, the United Kingdom, Switzerland, Norway, and Iceland in 2019. Due to the high volume of marketed PPPs, studying and monitoring these agricultural products thoroughly remains an essential task. PPPs are composed of at least one active substance, or pesticide, and various other compounds, called co-formulants, which determine the physicochemical properties of the mixture. While the active substances have been studied in depth and are strictly regulated, co-formulants added to PPPs are an emerging matter of concern.

In spite of the current lack of attention given to co-formulants, their possible toxicological effects have been reported. For instance, Straw et al.^2^ found that mortality in bees sprayed with a glyphosate-based PPP had a notably lower mortality rate than those exposed to a glyphosate-free PPP containing the same co-formulants, which clearly pointed at their co-formulants as potentially toxic compounds. Furthermore, it has also been determined that PPPs containing alkyl ethoxylates (AEOs), a common ingredient, are 100 times more toxic than PPPs not containing them.^3^ Other authors have concluded that toxicological effects of pesticides on common frogs can be highly dependent on the amount of co-formulants.^4^ Additionally, other authors have pointed at synergistic effects between the active substances and co-formulants, which could result in increased toxicity.^5,6^

Concerning EU Regulations, Regulation EC No 2021/383 lists the forbidden co-formulants for use in PPPs or marketed adjuvants.^7^ However, this regulation generally allows concentrations less than 0.1% (w/w) of these unacceptable co-formulants unless otherwise noted, as these could be unintentional impurities. It lists a total of 144 compounds, some of which are widely known for their potential toxicity, such as 4-nonylphenol, 4-octylphenol, and their isomers, which can act as endocrine disruptors.^8^ Other co-formulants included in this Regulation are solvents, petroleum distillates, borate derivatives, or asbestos fibers. At a national level, the Spanish Ministry of Health sets an additional list of additional unacceptable co-formulants.^9^

Studies focusing on the analysis of co-formulants by liquid chromatography (LC) are extremely scarce, as the few available studies usually address the determination of active substances, whereas those that consider co-formulants usually apply gas chromatography (GC).^10−14^ Despite this, previous studies have been carried out by other authors, with different methodologies and results. For instance, Schaller et al.^15^ carried out an extensive identification of 51 different PPPs including 10 emulsifiable concentrates (EC), 11 suspension concentrates (SC), 11 soluble concentrates (SL), 10 water-dispersible granules (WG), and 9 wettable powders (WP), marketed in Switzerland, resulting in the identification of a wide range of LC-amenable co-formulants. The used analytical technique was not disclosed. López-Ruiz et al.^16^ confirmed and quantified three co-formulants by LC-Exactive-Orbitrap-MS in three EC quizalofop-P-ethyl-based PPPs, by developing a strategy involving unknown analysis.

The main purpose of the present study is the tentative identification, confirmation, and quantification of LC-amenable co-formulants in PPPs authorized for their use in crops. This study also aims at the expansion of knowledge on the barely studied field of co-formulants used in PPPs, hoping to shed light on their real composition beyond their active substances, and to pave the way for further studies regarding the composition of PPPs. In all, 15 PPPs were characterized, including either difenoconazole (fungicide) or chlorantraniliprole (insecticide) as active substances. Samples were analyzed by means of ultrahigh-performance chromatography (UHPLC) coupled to high-resolution mass accuracy spectrometry (HRMAS). For this purpose, a Q-Exactive Orbitrap mass spectrometer was used, and data were acquired in full-scan MS and data-dependent acquisition (ddMS^2^) modes. Finally, a comprehensive data treatment strategy integrating suspect screening and unknown analysis was applied.

### Materials, Reagents, and Equipment

Fifteen chlorantraniliprole and difenoconazole-based PPPs of five types of formulations were acquired, and they are summarized in Table S1. These PPPs are Altacor 35WG (WG), Ampligo 150 (ZC), Ceremonia 25 EC (EC), Cidely Top (DC), Coragen 20 SC (SC), Dagonis (SC), Duaxo (EC), Dynali (DC), Kabuto JED (EC), Lexor 25 (EC), Mavita 250 (EC), Nomada (EC), Ortiva Top (SC), Score 25 EC (EC), and Voliam Targo (SC).

Regarding analytical-grade standards, 1,2-benzisothiazol-3(2*H*)-one (≥98.0%), hexaethylene glycol monotetradecyl ether (myreth-6) (≥99.0%), sodium dodecyl benzene sulfonate (CRM, 100%), and aniline (≥99.5%) were supplied by Sigma-Aldrich (St. Louis, MO). Sodium decyl sulfate (>98.0%) and 1-dodecylnaphthalene (>97.0%) were acquired from TCI (Zwijndrecht, Belgium). Naphthalene-1-sulfonic acid sodium salt was supplied by Alfa Aesar (99%), and lauramide DEA (≥95.0%) was purchased from Fluorochem (Hadfield, U.K.). SP Brij C2 (Ceteth-2) was purchased from Sigma-Aldrich just for confirmation purposes, as it is not an analytical standard.

Methanol (LC-MS Chromasolv, ≥99.9%), purchased from Honeywell (Charlotte, NC); water (LC-MS LiChromasolv), obtained from Merck (Darmstadt, Germany); and acetonitrile (LC-MS Chromasolv, ≥99.9%), supplied by Honeywell, were used to dissolve the PPPs or to prepare the mobile phase. Formic acid (LC-MS, 99.0%) was acquired from Fischer Scientific (Waltham, MD).

Samples were shaken in a vortex supplied by VWR International (Darmstadt, Germany). The chromatographic equipment was a Thermo Fisher Scientific Vanquish Flex Quaternary LC (Thermo Fisher Scientific), coupled to a Q-Exactive Orbitrap (Thermo Fisher Scientific) mass spectrometer. External mass calibration was performed by infusing a ProteoMass LTQ/FT-hybrid ESI mixture containing caffeine, acetic acid, Met-Arg-Phe-Ala-acetate salt, and Ultramark 1621 for ESI+ calibration. Regarding ESI calibration, an LTQ/FT-Hybrid ESI negative mixture including taurocholic acid sodium salt hydrate, sodium dodecyl sulfate, acetic acid, and Ultramark 1621 was infused. Mass-lock calibration was also performed.

### Sample Processing

Sample processing consisted of the dilution of the PPPs. PPPs were homogenized according to the procedure described by Vinke.^17^ For this purpose, packages up to 500 mL were shaken manually in all directions, while 1 L packages were shaken in a rotatory shaker, for 1 minute in both cases. Afterward, 40 μL aliquots of each PPP were diluted in 4 mL of LC-MS grade water, and the mixture was shaken vigorously for 1 min in a vortex mixer. This resulted in a 1:100 (v/v) dilution, which was further diluted. Thus, 100 μL of this mixture was dissolved in 450 μL of water and 450 μL of methanol, to give a 1000 (v/v) dilution. This last step was repeated, which resulted in a final dilution of 10,000 (v/v), which was analyzed by UHPLC-Q-Orbitrap-MS. Altacor 35, a WG formulation, is a solid product in the form of granules that must be first dissolved in water. Therefore, 2 g of Altacor were weighed and dissolved in 2 mL of LC-MS water, which yielded a 35% (w/v) chlorantraniliprole solution. This stock solution was then processed according to the procedure described for the other PPPs.

### LC-Q-Orbitrap-MS and LC-Q-Orbitrap-MS2 Conditions

Co-formulants were efficiently separated by UHPLC in a Hypersil GOLD aQ column (100 mm × 2.1 mm, 1.9 μm). Concerning chromatographic conditions, the mobile phase was composed of methanol as the organic phase, and an aqueous solution of formic acid (0.1%) as the aqueous phase, and it was pumped at a flow rate of 0.2 mL/min. The injection volume was 10 μL. Elution was carried out in gradient mode, according to the following profile: constant composition of 5% methanol from 0 to 2 min; increase up to 100% methanol from 2 to 16 min; constant composition of 100% methanol from 16 to 26 min; decrease to 5% methanol from 26 to 27 min, which was kept constant for another 3 min, to equilibrate the column. Thus, the total run time was 30 min.

Regarding detection, a Q-Exactive-Orbitrap analyzer was used. Acquisition was performed by full MS and ddMS^2^ in both positive and negative ionization modes. ESI conditions were: capillary temperature (300 °C), heater temperature (305 °C), N_2_ as sheath and auxiliary gas (95%), spray voltage (4 kV), and S-lens radio frequency (RF) level (50). Full-scan MS data were acquired in the *m*/*z* range 50–750, at a resolution of 70,000 at *m*/*z* 200, and an AGC target of 1e6. On the other hand, ddMS^2^ was carried out at a resolution of 35,000 at *m*/*z* 200 and an AGC target value of 1e5, loop count 5, and an isolation window of *m*/*z* 5.0. All data were acquired using the software Xcalibur Sequence Setup.

## Data Treatment Strategies

### Suspect Screening

Raw data for suspect screening were initially processed by an Xcalibur 3.0 Qual Browser. An extensive database containing 165 compounds was built from our findings,^16^ previous studies,^11,15,18^ and Regulation EC No. 2021/383,^7^ displayed in the first tab of Excel Sheet S1 in the Supporting Information. This database includes a wide range of co-formulants, starting from solvents, alkyl ethoxylates, preservatives, anionic and nonionic surfactants, perfluorinated compounds, alcohols, or nonylphenol and octylphenol derivatives, among many other types of compounds. These suspect compounds were then searched manually in all PPPs by their characteristic ions, either [M + H]^+^ or [M – H]^−^ adducts. Only suspected compounds with a mass error lower than 5 ppm, an acceptable peak shape, and undetected in blanks were taken into consideration. To check whether obtained fragments actually belonged to the parent compounds, experimental fragmentation results were compared with theoretical fragments for each compound. For this purpose, Mass Frontier 7.0 (Thermo Fisher Scientific) was used, as this software can predict all possible fragments, including their fragmentation path and provide their exact mass. Theoretical fragments were then obtained for all compounds and were matched with experimental ddMS^2^ fragments, considering a mass error lower than 5 ppm. Finally, analytical-grade standard of aniline was acquired and injected to confirm and quantify this putative co-formulant, according to its retention time, peak shape, and mass error.

### Unknown Analysis

Unknown analysis was carried out using Compound Discoverer 3.2 (Thermo Fisher Scientific), by means of 14 different ChemSpider libraries accounting for over 103 million compounds. Some of these libraries were generic, with many types of substances, while a few others focused on industrial additives including PPP co-formulants. These databases were: Alfa Chemistry, Alkamid, Aurora Fine Chemicals, EPA DSSTox, Chemspace, EPA Toxcast, FDA, FDA UNII–NLM, FooDB, KEGG, MassBank, Molbank, Nature Chemical Biology, and Nature Chemistry.

After the initial filters were applied, qualifying compounds were checked individually. The structure of every compound and their peak shape were visualized, and molecules neither matching with co-formulant-like compounds, with an irregular peak shape, nor with an excessively low S/N ratio, were not further considered. However, while automated data processing software is a helpful and powerful tool in the field of unknown analysis, peaks should also be checked manually to avoid possible errors like a missing or a poor peak integration, as it has been suggested by other authors.^19^ Moreover, every Compound Discoverer entry can be associated with multiple compounds just ranked by their number of citations, so all of them also need to be checked under the same criteria. After Compound Discoverer search concluded, a literature review was carried out to determine whether the remaining compounds could be compatible with PPPs, including previous reports of their addition to PPPs. Co-formulants suspected of being used in PPPs were studied in Xcalibur. This software can predict isotopic patterns, displaying mass spectra as well as chromatograms.

Finally, putative confirmation was carried out in agreement with ddMS^2^ data for all final compounds, according to the classification system for the identification of compounds proposed by Schymanski et al.,^20^ which is based on different confidence levels. Analytical standards of 1,2-benzisothiazol-3(2*H*)-one, naphthalene sulfonate, sodium decyl sulfate, 1-dodecylnaphthalene, lauramide DEA, sodium dodecyl benzene sulfonate, and myreth-6 were then acquired and injected to confirm and quantify them. Retention time, peak shape, and MS^2^ spectra were the main criteria that led to the confirmation of the co-formulants.

## Results and Discussion

### Dilute and Shoot Sample Optimization

The solubility of PPPs in acetonitrile, methanol, and water was tested to choose the most suitable solvent. For this purpose, 40 μL of each PPP were spiked in 4 mL of each solvent. In all cases, water was the only solvent compatible with the studied technical formulations. It was also observed that highly hydrophilic components would lump together and form an insoluble white precipitate in the organic solvents, meaning that analytes would be lost during the dilution process. This finding is related to the fact that PPPs have been developed and formulated in such a way that they can be dissolved entirely in water since their on-field application is meant to be done by diluting the PPP in water. Therefore, using water ensured that the analyte loss would not take place.

Once the solvent was chosen, several dilutions were tested. After PPPs were initially dissolved in water, they were diluted with methanol:water 50:50 (v/v), which was the mixture used in all prepared dilutions. In this case, no precipitate or signs of inhomogeneity were observed. As PPPs contain active substances at high concentrations, up to 35% (v/v) in our samples, they must be diluted prior to injection, which will reduce the likeliness of contamination in the analytical equipment. Therefore, a balance between the amount of analyte and the cleanliness of the equipment must be found, to ensure that analytes can be correctly detected and identified, while avoiding contamination to the extent possible. Studied dilutions were: 1:1000 (v/v), 1:10,000 (v/v), 1:100,000 (v/v), and 1:1,000,000 (v/v). Samples of each PPP were assessed by comparing the number of identified compounds. Overall, better peak shapes were obtained for dilutions 1:1000 (v/v) and 1:10,000 (v/v). The number of results in unknown analysis was also assessed. Dilution 1,000,000 (v/v) proved insufficient for unknown analysis, as no results were obtained for any sample, so it was ruled out. It is important to note that while higher dilutions such as 1,000,000 (v/v) may work for manual suspect screening carried out by an experienced analyst, lower dilutions are required for unknown analysis, to ensure that the nontarget software limitations regarding correct ion detection at low concentrations can be overcome. However, at lower dilutions, most of the provided results by ion-detecting software refer to detected blank compounds, which could be misleading at first. Therefore, the authors of this paper encourage the manual verification of all results, especially if the injected sample is highly diluted. Despite this, several dilutions had to be injected for quantitative purposes for some PPPs since the concentration of identified co-formulants greatly differs within the same PPP.

### LC-Q-Orbitrap Suspect Screening

A total of 12 compounds were tentatively identified by suspect screening, as shown in Table 1. Four of these co-formulants are currently banned by Regulation EC No 2021/383,^7^ whereas 1 of them is banned by the Spanish Ministry of Health.^9^ Banned compounds were: 2-[2-[4-(1,1,3,3-tetramethylbutyl)phenoxy]ethoxy]ethanol,17-(4-nonylphenoxy)-3,6,9,12,15-pentaoxaheptadecan-1-ol, 20-(4-nonylphenoxy)-3,6,9,12,15,18-hexaoxaicosan-1-ol, 1-methylpyrrolidin-2-one, and aniline. However, analyzed PPPs were marketed in Spain prior to the enforcement of this legislation, and a certain concentration must be met so that the presence of such co-formulant in the PPP can be considered unacceptable.

**Table 1:** Co-formulants Tentatively Identified by Suspect Screening^a^

	Ceremonia 25 (EC)	Duaxo (EC)	Kabuto JED (EC)	Lexor 25 (EC)	Mavita 250(EC)	Nomada (EC)	Score 25(EC)	Coragen 20 (SC)	Dagonis (SC)	Ortiva Top (SC)	Voliam Targo (SC)	Cidely Top (DC)	Dynali (DC)	Ampligo 150 (ZC)	Altacor 35WG (WG)
2-palmitoylglicerol	Yes	Yes	Yes	Yes	Yes	Yes	Yes	Yes	Yes	Yes	Yes	Yes		Yes	
9-octadecenamide		Yes	Yes												
glyceryl monostearate	Yes	Yes	Yes	Yes	Yes	Yes	Yes	Yes	Yes	Yes	Yes	Yes		Yes	
2-[2-[4-(1,1,3,3-tetramethylbutyl)phenoxy]ethoxy]ethano^b^^,^^d^		Yes	Yes												
17-(4-nonylphenoxy)-3,6,9,12,15-pentaoxaheptadecan-1-ol^b^^,^^d^				Yes	Yes		Yes								
20-(4-nonylphenoxy)-3,6,9,12,15,18-hexaoxaicosan-1-ol^b^^,^^d^				Yes			Yes								
aniline^c^								Yes			Yes			Yes	Yes
dipropylene glycol methyl ether		Yes	Yes												
methylchloroisothiazolinone								Yes							
*N*,*N*-dimethyldecanamide	Yes											Yes	Yes		
1-methylpyrrolidin-2-one^b^^,^^d^				Yes	Yes		Yes			Yes		Yes	Yes		
nonaethylene glycol monododecyl ether^d^	Yes			Yes	Yes		Yes	Yes							

a Abbreviations: DC: dispersible concentrate; EC: emulsifiable concentrate; SC: suspension concentrate; WG: wettable granules; ZC: a mixture of capsule suspension (CS) in SC.

b Banned according to EC Regulation No 2021/383.

c Banned according to the Spanish Ministry of Health.

d Formed [M + Na]^+^ adducts.

**Figure 1:**
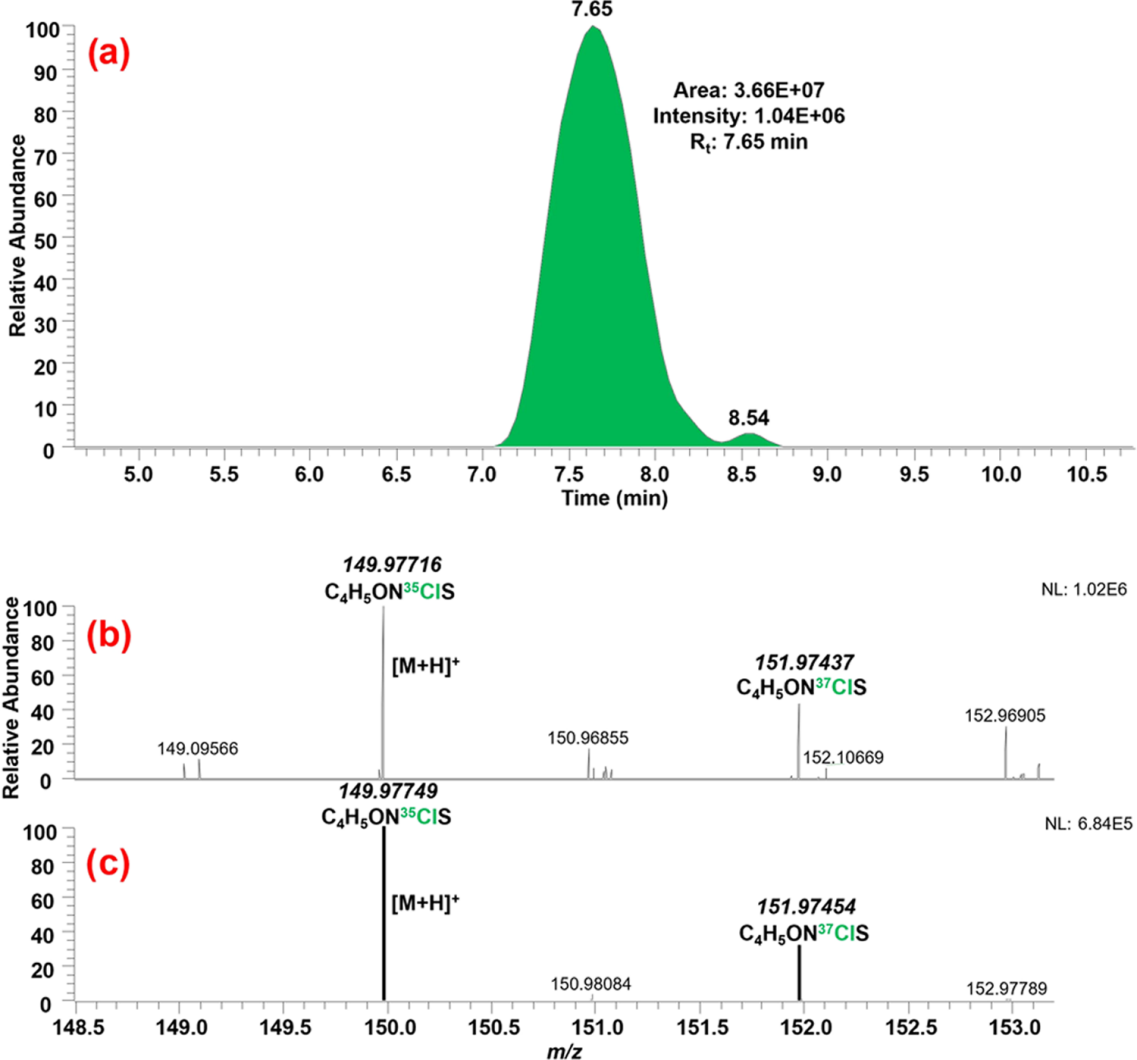
Tentative identification of methylchloroisothiazolinone in Coragen 20 SC by isotopic pattern: (a) chromatographic peak; (b) full-scan MS spectrum, and (c) predicted MS spectrum and isotopic pattern.

Figure 1 shows the tentative identification of methylchloroisothiazolinone in Coragen 20 SC by comparing the experimental full-scan MS and predicted spectra. A Gaussian peak can be observed at 7.65 min in the EIC chromatogram, when the ion *m*/*z* 149.97749 was monitored. The full-scan MS spectrum revealed that ion *m*/*z* 149.97716 was the [M + H]^+^ adduct, with a mass error of −2.20 ppm. A closer look also evidenced the presence of the chlorine isotopic pattern, in which the ^35^Cl and ^37^Cl isotopes can be seen, with an abundance of approximately 100 and 35%, respectively, which matched the abundance of the predicted MS spectrum.

Some compounds also formed [M + Na]^+^ adducts in addition to the characteristic [M + H]^+^ ones. In some cases, sodium adducts were even more abundant than the protonated adducts. This seems to be a relatively frequent phenomenon for the analyzed alkyl ethoxylates.^21^ On the other hand, all compounds exhibited an adequate peak shape, and mass error was lower than 5 ppm in all cases. However, relying only on characteristic ions can become a hurdle in suspect screening, since detecting these characteristic ions does not ensure the presence of these suspected co-formulants. To deal with this issue, at least two fragments are required for a proper putative identification, in accordance with SANTE guidelines.^22^ Nonetheless, full-scan MS data seldom provide any fragment ion, and as a consequence of it, full-scan MS data are not enough for reliable putative identifications. Therefore, data-dependent acquisition (ddMS^2^), which can provide results comparable to those from multiple reaction monitoring (MRM) acquisition, was used to induce the fragmentation of the characteristic ion so that the combination of full-scan MS and ddMS^2^ could allow the distinction of different structural isomers. This was the case for butyl glycol, which was initially identified based on its characteristic ion *m*/*z* 119.10666, although the presence of this compound could later be ruled out thanks to ddMS^2^, since none of the experimental and its predicted ddMS^2^ fragments matched. Thus, two fragments were searched for every compound by ddMS^2^, and compounds reaching this stage were considered suitable for comparison with analytical standards

**Figure 2:**
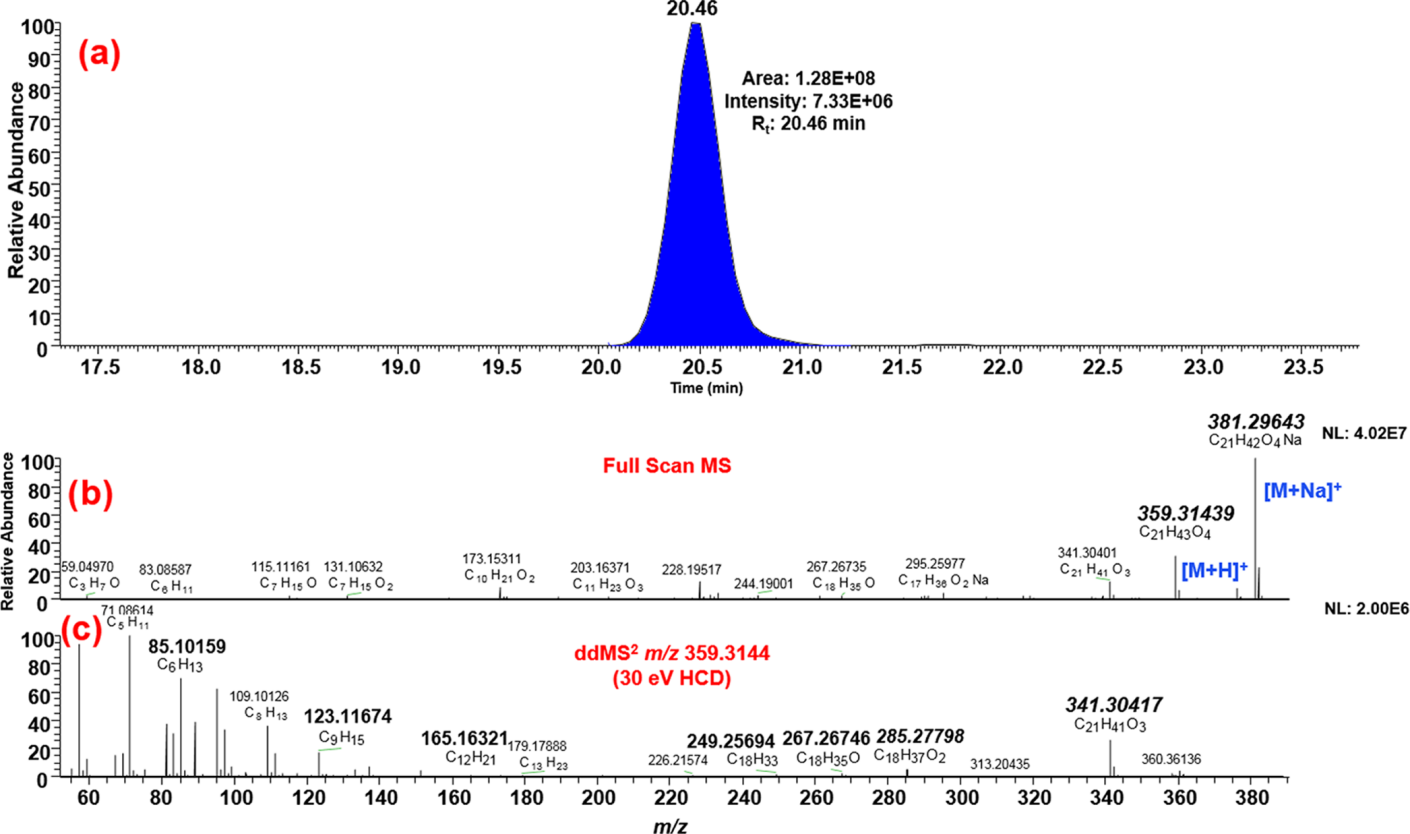
Tentative identification of glyceryl monostearate in ddMS^2^: (a) chromatogram; (b) full-scan MS spectrum showing [M + H]^+^ and [M + Na]^+^ adducts, and (c) ddMS^2^ spectrum of ion *m*/*z* 359.3144 at 30 eV.

Figure 2 shows tentative identification of glyceryl monostearate by ddMS^2^ data. Theoretical fragments provided by Mass Frontier (341.30502; 285.27881; 267.26824; 249.25768; 165.16378; 123.11683 and 85.10118) were found in the ddMS^2^ spectrum, with a maximum mass error of −2.79 ppm. Considering the large number of reported matching fragments, and the fact that the first four of them have a considerably high *m*/*z* value compared to the characteristic ion, which makes them really reliable for tentative identification, it can be concluded that glyceryl monostearate has been tentatively identified. It is important to note that ddMS^2^ and isotopic pattern are complementary tools. For instance, the tentative identification of a suspected co-formulant containing S, Cl, Br, or I can be dismissed without having to resort to ddMS^2^ fragmentation, by just looking for its isotopic pattern; the lack of any isotopic pattern in the MS spectrum automatically confirms that the real co-formulant cannot contain any of these heteroatoms. However, ddMS^2^ usually remains the preferred criteria for tentative identification due to its high reliability. Therefore, the isotopic pattern should be used along ddMS^2^ data for a satisfactory tentative identification, unless ddMS^2^ data are unavailable, in which case a less confident tentative identification could be carried out by only considering the isotopic pattern, if any, and the characteristic ion. To sum up, tentative identification will be more reliable if more tools are used.

### LC-Q-Orbitrap Unknown Analysis

Applied Compound Discoverer search parameters for unknown analysis were: mass error lower than 5 ppm, formation of [+H]^+^, [M − H]^−^ or [M + Na]^+^ adducts, 30% intensity tolerance, minimum peak intensity of 1,000, and S/N threshold of 3. Unnamed detected compounds were then ruled out, which narrowed the total number of possible compounds. Subsequently, various filters were applied, such as a mass error under 5 ppm, formulas containing only C, H, O, N, P, or S, and peak area in sample no less than 1e6 counts, and no peak area in any blank. This shortened the total number of compounds even more, but over 1000 possible entries could still qualify.

**Figure 3:**
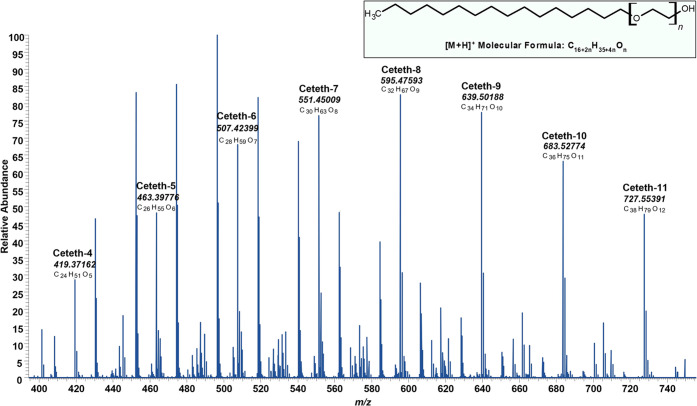
Characteristic full-scan MS spectrum of several ceteth alkyl ethoxylates in Lexor 25.

This unknown analysis helped identify 66 co-formulants, as shown in Table S2, and were added to the homemade database. Some of these tentatively identified compounds were polyethylene glycols (PEG), nonionic surfactants such as alkyl ethoxylates (ceteth, trideceth, myreth, steareth, or oleth derivatives), anionic surfactants, such as C9-C14 linear alkylbenzene sulfonates (LAS), emulsifiers (PEG-4 sorbitan stearate), dispersants (linear and branched naphthalene sulfonates), antistatic agents (lauryldiemthylamine oxide), amine and amide surfactants (Lauramide DEA, castor oil diethanolamide), stabilizers and preservatives including biocides (1,2-benzisothiazol-3(2*H*)-one), crystal growth inhibitors (*N*,*N*-diethyloctanamide) or antioxidants (metilox), among many other types of compounds. Alkyl ethoxylates were the most recurrent co-formulants by far. Alkyl ethoxylates are not added to PPPs as a single compound, but rather as a mixture of multiple polymers, formed by adding subunits of the monomer ethylene glycol to the main chain. This is tightly related to their synthesis, which involves the use of mixtures of different PEG molecules. Figure 3 depicts the full-scan MS spectra of different ceteth (polyethylene glycol monohexadecyl ether) polymers in the PPP Lexor 25, ranging from ceteth-4 to ceteth-11. It could be observed that these alkyl ethoxylates present very characteristic bell-shaped MS spectra, in which each *m/z* peak corresponds to an ethoxylated polymer, separated by *m*/*z* 44.02567, that corresponds to the added C_2_H_4_O subunits, as shown in the structure. These features allow for an easy and quick identification of polyethoxylates in samples by LC-MS. Moreover, all of these compounds produce a single chromatographic peak, which means that they cannot be separated under standard chromatographic conditions, and hence, a special chromatographic column would be required for their separation. This behavior has also been previously described in polyoxyethylene tallow amine surfactants.^23^ Another way of tentatively identifying possible co-formulants in PPPs is the manual revision of the total ion chromatogram (TIC) and mass spectrum, which will complement the search of compounds that are unavailable in ChemSpider databases. Data-processing software can provide a list of possible molecular formulas for any selected *m*/*z* value, based on several filters such as type and number of atoms or maximum mass error, and these molecular formulas are then searched in other online libraries such as PubChem. A list of possible compounds matching those molecular formulas is obtained, which requires further literature review to assess whether these compounds can have co-formulant-like properties or are likely to be used in PPPs. This works especially well for co-formulants with unique molecular formulas, such as either long-chain co-formulants or those containing heteroatoms, as there will be fewer potential candidates. These candidates are then subjected to ddMS^2^ analysis, according to the previously explained strategy.

### Presence of Co-Formulants in PPPs

**Figure 4:**
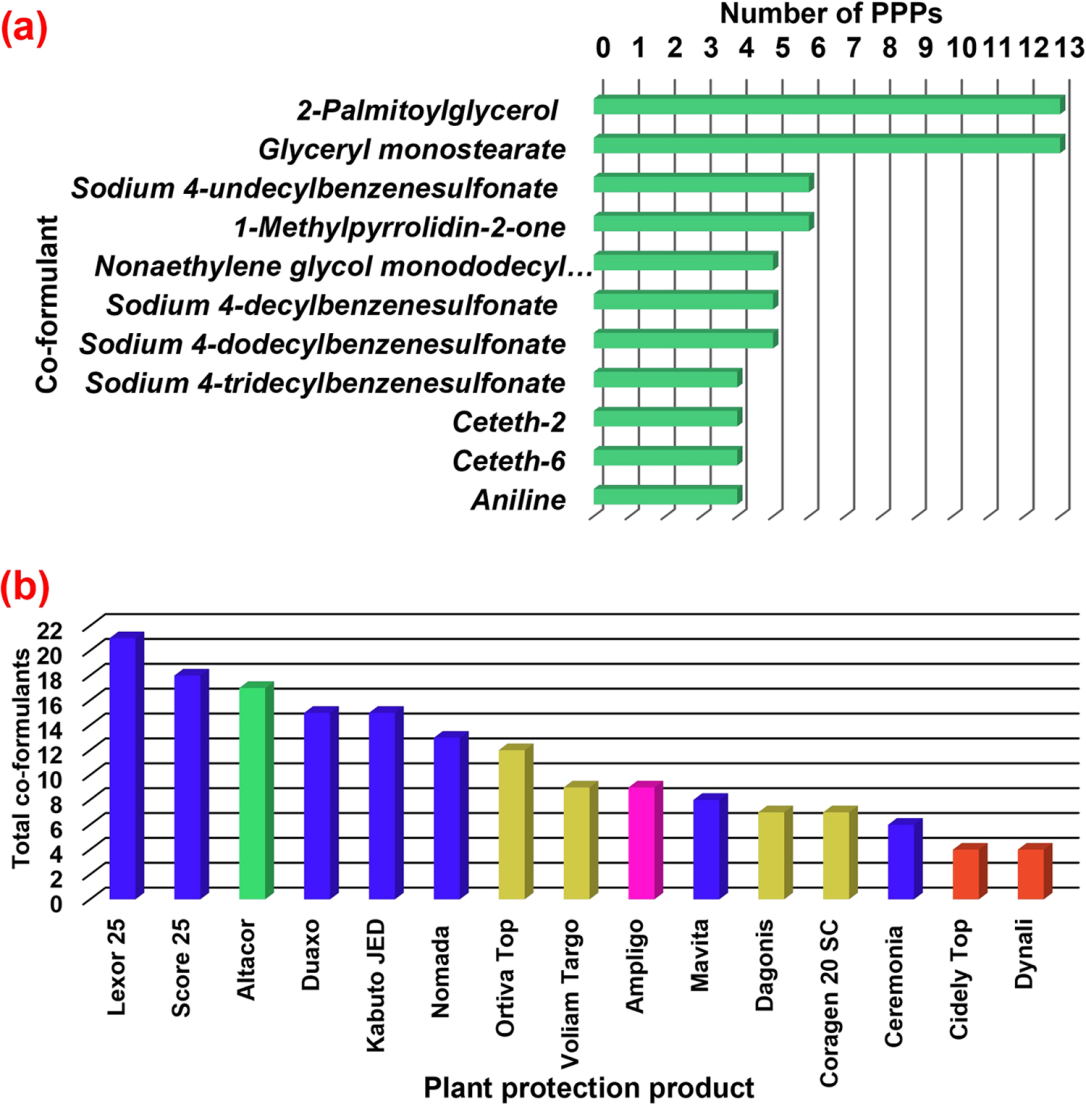
Presence of co-formulants in PPPs: (a) number of PPPs containing the most recurrent co-formulants and (b) total number of co-formulants identified in each PPP.

In all, 78 compounds were tentatively identified in 15 PPPs (EC, DC, SC, WG, and ZC); 12 by suspect screening; and 66 by unknown analysis, all of which eluted from 1.27 to 24.57 min. Table S2 shows the presence of co-formulants in each studied PPP, whereas Figure 4a represents the number of PPPs containing the most recurrent co-formulants. 2-Palmitoylglycerol and glyceryl monostearate are by far the most common co-formulants, present in all PPPs except for 2 formulations: Dynali (DC) and Altacor (WG), followed by sodium 4-undecylbenzenesulfonate and 1-methylpyrrolidin-2-one, both detected in six PPPs. Nonaethylene glycol monododecyl ether was identified in five samples (4 EC and 1 SC), like sodium 4-decylbenzenesulfonate (3 EC, 1 SC, and 1 ZC) and sodium 4-dodecylbenzenesulfonate (3 EC, 1 SC, and 1 ZC). Finally, sodium 4-tridecylbenzenesulfonate, ceteth-2, ceteth-6, and aniline were detected in four PPPs. Many other compounds were only detected in a single PPP, which suggests a huge diversity in composition. Moreover, different alkyl ethoxylates were identified in 9 out of 15 analyzed PPPs, which makes the most common family of co-formulants in this study.

Regarding the individual composition of PPPs, some interesting observations can be inferred. As Figure 4b shows, Lexor 25 (EC) PPP had the greatest amount of co-formulants, with up to 21 identified compounds, followed closely by Score 25 (EC) and Altacor (WG), with 18 and 17 co-formulants, respectively. Duaxo and Kabuto JED, both EC formulations, presented 15 compounds. Interestingly, Kabuto JED (EC) and Duaxo (EC) have an identical composition according to Table S2, which suggests a common manufacturer, even though both products are marketed under different brands. Other formulations were: Nomada (EC, 13 co-formulants), Ortiva Top (SC, 12 co-formulants), Voliam Targo (SC, 9 co-formulants), Ampligo (ZC, 9 co-formulants), Mavita (8 co-formulants), Dagonis (SC, 7 co-formulants), Coragen 20 SC (7 co-formulants), Ceremonia (EC, 6 co-formulants), Cidely Top (DC, 4 co-formulants), and Dynali (DC, 4 co-formulants).

To sum up, EC formulations had an average of 13 identified co-formulants per PPP, whereas SC formulations had an average value of almost 9 identified co-formulants per PPP. Contrary to this, the two analyzed DC formulations showed few to no LC-amenable co-formulants, with an average of barely four identified co-formulants per PPP, making it the type of formulation with the lowest amount of identified co-formulants. This matches our previous findings on GC-amenable co-formulants, suggesting that EC formulations have by far the greatest number of co-formulants.^10^

However, no general relation could be established between the type of formulation and the type of substances found, as many of these substances could be found in up to four different formulations. However, Altacor presented the most unique composition compared to other PPPs, and no co-formulant identified in Altacor was found in any other PPP, which is probably due to the fact that it is a solid formulation while the other analyzed PPPs are liquid formulations (EC, DC, SC, and ZC). Therefore, it seems likely that differences on the physical state of PPPs may have a more direct impact on its composition, rather than the specific type of formulation. Additionally, no significant differences were observed between chlorantraniliprol-based and difenoconazole-based PPPs.

### Confirmation

The performed identification was merely putative, so analytical standards must be used to confirm the detected compounds, by means of retention times, peak shapes, and matching MS spectra. Since not many analytical standards of identified compounds were available on the market, a few of them were acquired, with a special focus on those identified on Altacor 35WG, due to the high number of different tentatively identified compounds.

A total of nine commercially available analytical standards were then purchased, one of which corresponded to a co-formulant identified by suspect screening (aniline), and eight to co-formulants identified by unknown analysis (Table 2). Subsequently, these analytical standards were injected so as to carry out the confirmation of tentatively identified co-formulants and their quantitation. All nine compounds were confirmed successfully, and no false positives were detected during the confirmation stage, which hints at the high reliability of the described analytical methodology for the tentative identification of co-formulants.

**Table 2:** Quantitative Results of Co-Formulants in Different PPPs (g/L)^a^^,^^b^

	Ceremonia 25 (EC)	Duaxo (EC)	Kabuto JED (EC)	Lexor 25 (EC)	Mavita (EC)	Nomada (EC)	Score 25 (EC)	Coragen 20 (SC)	Ortiva Top (SC)	Voliam Targo (SC)	Ampligo 150 (ZC)	Altacor 35WG (WG)
1,2-benzisothiazol-3(2*H*)-one										0.20	0.24	
aniline										0.05		190.01
naphthalene sulfonate												222.82
sodium decyl sulfate												0.70
1-dodecylnaphthalene												8.45
lauramide DEA												1.11
sodium dodecyl benzene sulfonate	26.33	10.35	11.67	28.15	32.33	16.93	28.30				0.83	
myreth-6				0.03				0.17				
ceteth-2^c^				NA	NA	NA	NA		NA			

a Abbreviations: DC: dispersible concentrate; EC: emulsifiable concentrate; NA: not available; SC: suspension concentrate; WG: wettable granules; ZC: a mixture of capsule suspension (CS) in SC.

b Altacor results are expressed in μg/g.

c Confirmed, but not quantified, since the purchased ceteth-2 standard did not meet the minimum criteria for quantification.

**Figure 5:**
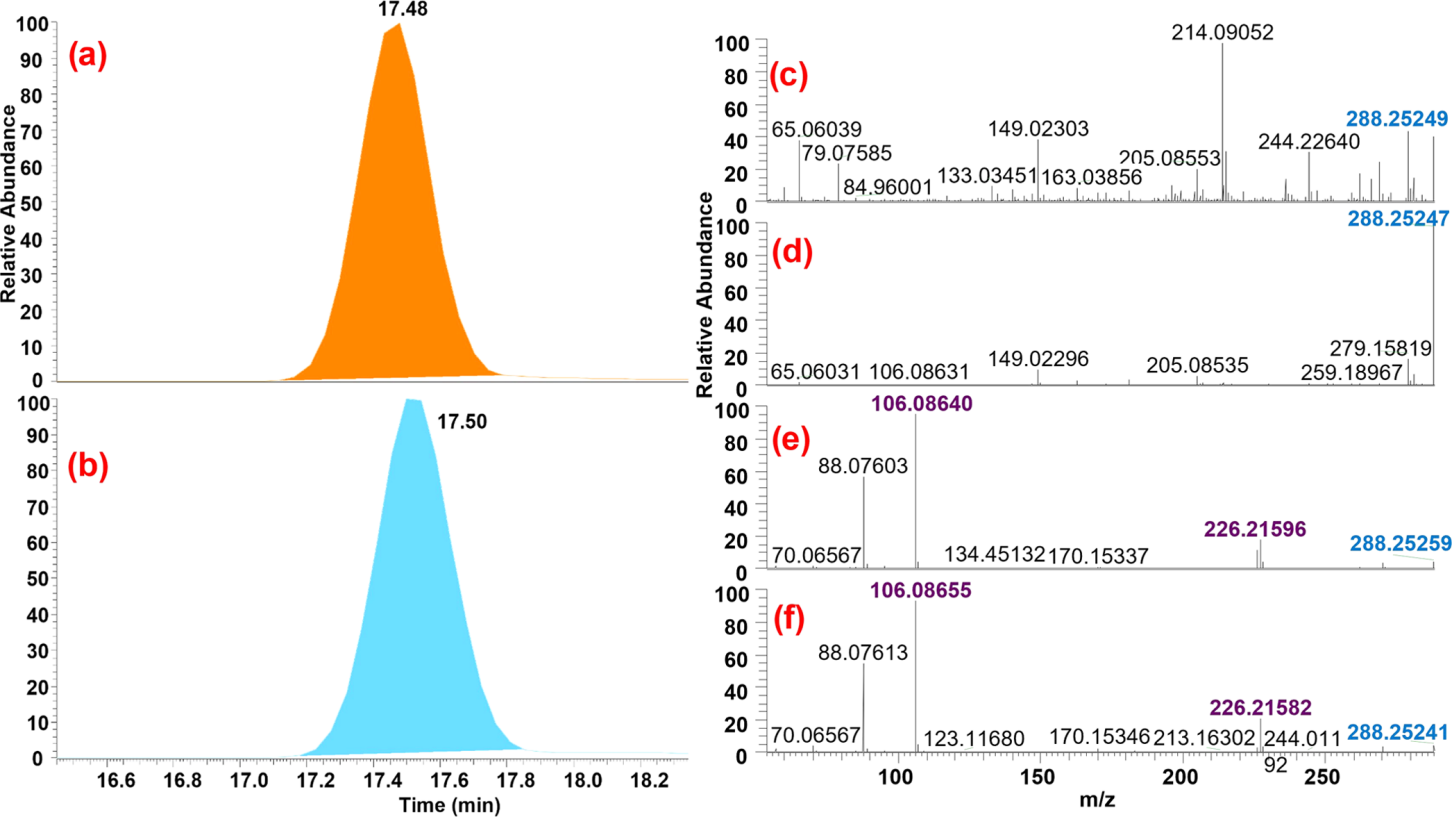
Confirmation of lauramide DEA: (a) altacor chromatogram; (b) analytical standard chromatogram (20 μg/L); (c) full-scan MS spectrum of the sample; (d) full-scan MS spectrum of the analytical standard; (e) ddMS^2^ spectrum of ion *m*/*z* 288.25332 in Altacor, and (f) ddMS^2^ spectrum of ion *m*/*z* 288.25332 in the analytical standard.

Figure 5 shows the confirmation of lauramide DEA in Altacor via chromatograms, full-scan MS spectra, and ddMS^2^ spectra. The retention time shift was 0.02 min, lower than 0.1 min. In this case, the mass error for the characteristic ion 288.25332 was −2.9 ppm. ddMS^2^ spectra also showed a highly matching pattern, while smaller differences were due to the matrix interferences in the sample. Fragments at *m*/z 226.21654 and 106.08626 had a mass error of −2.6 ppm and 1.32 ppm, respectively.

After these co-formulants were confirmed by means of analytical standard, literature research was carried out to clarify the function of each of them. This way, 1,2-benzisothiazol-3(2*H*)-one was found to be a biocide used in PPPs.^24^ Naphthalene sulfonate is an anionic wetting and dispersing agent added to PPPs, which also has stabilizing properties.^25^ Sodium decyl sulfate is another anionic co-formulant, which has been reported to act as a surfactant in PPPs, like many other alkyl sulfates, most notably sodium dodecyl sulfate (SDS).^26^ Furthermore, lauramide DEA is a thickener, foam booster, and stabilizer widely used in cosmetics and shampoos due to its properties, but there is no previous literature on its use in PPPs.^27^ Ceteth-2 is an alkyl ethoxylate, which is included in PPPs due to its defoaming, emulsifying, antistatic, wetting, and solubilizing properties.^28^ Myreth-6 is also an alkyl ethoxylate, and as such, it shares properties with ceteth-2. However, the utility of 1-dodecylnaphthalene and aniline in PPPs remains unknown, even though the use of aniline in agricultural fungicides and herbicides has been reported.^29^ Thus, these findings justify the presence of these co-formulants in the analyzed PPPs and increase the confidence in the presented results.

### Method Validation and Quantitation

After confirmation of nine different co-formulants by analytical standards, their quantitation was carried out, except for ceteth-2. The main hurdle was the lack of any blanks and the complexity of the mixtures, so matrix-matched calibration standards could not be prepared. Instead, the standard addition methodology was applied, in which samples were spiked with a standard solution containing all analytes. Nonetheless, the applied method had to be validated in accordance with SANTE/11312/2021 guidelines,^22^ which ensured reliable quantitative results. The assessed validation parameters were linearity, matrix effect, specificity, interday precision (RSD), limit of quantification (LOQ), and retention time. It is important to note that recoveries should not be determined for the direct analysis of liquid samples, as in this case, and according to SANTE guidelines, only the precision should be evaluated by calibration standards. While Altacor 35WG is a solid formulation, it dissolves entirely in water, so recoveries were not calculated either as it cannot be considered an extraction per se. PPPs were carefully selected so that validation could be performed representatively in four types of formulations containing all confirmed co-formulants (EC, SC, WG, and ZC), and all analytes could be assessed. Thus, Lexor 25 (EC), Voliam Targo (SC), Altacor (WG), and Ampligo (ZC) were chosen.

Table 3 shows all of the determined validation parameters. Standard addition and solvent calibration standards were prepared from 1 μg/L to 600 μg/L, with all *R*^2^ values being greater than 0.9994. No matrix effect was appreciated (ME < 20%) for any co-formulant. Furthermore, interday precision (% RSD) at 100 μg/kg (Altacor) and 100 μg/L (liquid formulations) was lower than 8% in all cases, and retention time remained constant, with a shift lower than 0.1 min. Finally, the lowest LOQ obtained was 0.001 g/L for liquid formulations (1,2-benzisothiazol-3(2*H*)-one), and 0.0001 mg/g for the solid formulation (sodium decyl sulfate). Therefore, the method was successfully validated.

**Table 3:** Validation Parameters of Confirmed Co-Formulants^a^

co-formulant	PPP	matrix effect (%)	interday precision^b^ RSD (%)	linearity *R*^2^	method LOQ
1,2-benzisothiazol-3(2*H*)-one	Voliam Targo (SC)	–13	3	0.9998	0.001 g/L
aniline	Altacor (WG)	16	4	0.9984	0.05 mg/g
naphthalene sulfonate	Altacor (WG)	6	2	0.9998	0.001 mg/g
sodium decyl sulfate	Altacor (WG)	1	4	0.9998	0.0001 mg/g
1-dodecylnaphthalene	Altacor (WG)	9	8	0.9997	0.008 mg/g
lauramide DEA	Altacor (WG)	1	5	0.9976	0.0005 mg/g
sodium dodecyl benzene sulfonate	Ampligo (ZC)	20	2	0.9977	0.05 g/L
myreth-6	Lexor 25 (EC)	12	5	0.9980	0.005 g/L

a Abbreviations: LOQ: Limit of quantification; PPP: plant protection product; RSD: relative standard deviation.

b Interday precision calculated at 100 μg/kg (Altacor) and 100 μg/L (liquid PPPs).

Afterward, co-formulants were quantified using the solvent calibration curve, as there was no matrix effect. Results are shown in Table 2. Ceteth-2 could not be quantified as the purchased standard was not an analytical-grade standard. Sodium dodecyl benzene sulfonate, a linear alkylbenzene sulfonate, stands out as the most abundant co-formulant, with a value of up to 32.33 g/L in Mavita (EC). This compound has similar concentrations in the other five EC formulations in which it has been detected, ranging from 10.35 g/L in Duaxo to 28.30 g/L in Score 25. Interestingly, its concentration in Ampligo, a ZC formulation, was as low as 0.83 g/L, which is roughly 12 times smaller than the lowest concentration determined in an EC formulation (10.35 g/L). On the other hand, the lowest quantified co-formulant in any liquid formulation was myreth-6 (0.03 g/L), in Lexor 25 (EC), 0.003% (w/v), whereas its concentration in Coragen 20 SC (SC) was 0.17 g/L. The biocide 1,2-benzisothiazol-3(2*H*)-one was found at similar concentrations in Voliam Targo (SC) and Ampligo (ZC), with values of 0.20 and 0.24 g/L, respectively. Finally, aniline was quantified at 0.05 g/L in Voliam Targo.

Concerning Altacor (WG), a solid formulation, five co-formulants were quantified. Naphthalene sulfonate and aniline were by far the most concentrated co-formulants, 222.82 and 190.01 μg/g, respectively, in contrast to 1-dodecylnaphthalene (8.45 μg/g), lauramide DEA (1.11 μg/g), and sodium decyl sulfate (0.70 μg/g). Therefore, the lowest achieved quantification in the solid formulation was 0.00007% (w/w). These findings confirm that our analytical strategy allows for the detection and quantification of co-formulants present in commercial PPPs in concentrations as low as 0.00007% (w/w) and 0.003% (w/v). Additionally, aniline, the only quantified co-formulant considered unacceptable, was not found to be present in a concentration higher than the maximum stipulated to be considered an unintentional impurity, set in 0.1% (w/w).

### Risk Assessment Studies

As PPPs are added to the widely consumed agricultural commodities, humans can be exposed in multiple ways to co-formulants. Some of them include oral intake, inhalation, or skin absorption. Thus, toxicological information is required to assess whether the most commonly found co-formulants can pose a threat to human health. Currently, the main toxicological parameters are oral reference dose (RfD), reference concentration (RfC), and non-observed-adverse-effect level (NOAEL).^30^ However, there is little literature on the toxicological properties of the identified co-formulants, in contrast to active substances. The few available studies usually address the toxicological assessment of complex families of co-formulants, rather than specific co-formulants. This is the case of alkyl ethoxylates, alkylbenzene sulfonates, or alkylnaphthalene sulfonates, which can have multiple compounds.

The available toxicological information of the confirmed co-formulants and most representative substances are gathered in Table S3. Toxicological data for sodium decyl sulfate was unavailable, so information regarding sodium dodecyl sulfate (SDS), a very similar co-formulant, was reviewed instead. It can be observed that SDS showed the lowest toxicity according to its RfD value (1 mg/kg/day). On the other hand, its concentration in Altacor was only 0.70 μg/g, so no toxicological concern should rise from the addition of this specific surfactant to Altacor. Alkylbenzene sulfonates, which encompass many of the co-formulants tentatively identified in this study and a confirmed co-formulant, had a similar, but lower, oral RfD (0.5 mg/kg/day). The high concentrations of alkylbenzene sulfonates in the analyzed PPPs, up to 3.23% (w/v), could be compensated with an RfD value greater than many of the other studied co-formulants; 29 times greater than 1,2-benzisothiazol-3(2*H*)-one (0.017 mg/kg/day) and 71 times greater than aniline (0.007 mg/kg/day), which had the smallest RfD value, and thus, it is considered the most toxic analyzed co-formulant. Additionally, on average, the concentration of sodium dodecyl benzene sulfonate was 100 times higher than 1,2-benzisothiazol-3(2*H*)-one and 440 times higher than aniline. So, it confirms that the health risk associated with the intake of alkylbenzene sulfonates from PPPs is lower than that of the other two co-formulants. Alkylnaphthalene sulfonates also had an RfD value of 0.5 mg/kg/day, which technically makes them as toxic as alkylbenzene sulfonates, which is reasonable, considering that both families of anionic surfactants share a closely similar structure.

Finally, the literature was reviewed for ceteth, a common group of alkyl ethoxylates, which was tentatively identified in five different PPPs. No information regarding their RfD was found. However, the oral median lethal dose (LD_50_) values were reported in rats for ceteth-2, ceteth-10, and ceteth-20, the first of which was confirmed, and the second of which was tentatively identified in this study. Ceteth-2 stands out as the least lethal alkyl ethoxylate, with a value greater than 25.1 g/kg, followed by ceteth-20 (3.59 g/kg) and ceteth-10 (2.5 g/kg). Overall, the authors of that study consider it to be safe for human use, although they warn against their hypothetical degradation to ethylene oxide and 1,4-dioxane, two oxidation products.^28^

In conclusion, this study resulted in the tentative identification of 78 co-formulants and the confirmation via analytical standards of 9 of them in 15 PPPs (DC, EC, SC, WG, and ZC). UHPLC-HRMAS, a cutting-edge analytical technique, was successfully applied, following a hybrid data treatment strategy combining suspect screening and unknown analysis, for a comprehensive assessment on the presence of co-formulants in PPPs. The use of HRMAS, as opposed to previous studies focusing on either low-resolution mass spectrometry or other detection techniques, provided a reliable tentative identification through mass accuracy and ddMS^2^ data, which are not available in conventional LC-MS techniques. It is important to note that toxicological properties of the most common co-formulants in formulations for agricultural commodities should not be underestimated as they may involve health risks since they are likely to be a part of the food chain. The lack of information regarding the composition of PPPs does not suit the high volume of sales of PPPs and calls for more studies focusing on the study of the co-formulants contained on these ubiquitous technical formulations. Finally, the proposed methodology could be used in further studies where co-formulant residues can be determined in crops and environmental samples, providing a thorough insight into the real extent of the presence of these compounds in those samples after the application of PPPs.

## Supporting Information Available

Analyzed difenoconazole and chlorantraniliprole-based commercial PPPs (Table S1); tentatively identified compounds in analyzed PPPs (Table S2); and toxicological information of identified co-formulants (Table S3) (PDF)
