# Supplementary material for: Assessment of Co-Formulants in Marketed Plant Protection Products by LC-Q-Orbitrap-MS: Application of a Hybrid Data Treatment Strategy Combining Suspect Screening and Unknown Analysis
Source: J Agric Food Chem. 2022 Jun 7;70(23):7302–13. doi: 10.1021/acs.jafc.2c01152 (PMC9204808; doi:10.1021/acs.jafc.2c01152)
Supplement: Supplementary file 1 — jf2c01152_si_001.pdf [file jf2c01152_si_001.pdf]

SUPPORTING INFORMATION to the article: Assessment of co-formulants in marketed plant protection products by LC-Q-Orbitrap-MS: application of a hybrid data treatment strategy combining suspect screening and unknown analysis

Antonio Jesús Maldonado-Reina, Rosalía López-Ruiz, Roberto Romero-González, José Luis Martínez Vidal, Antonia Garrido-Frenich*

Research group "Analytical Chemistry of Contaminants", Department of Chemistry and Physics, Research Centre for Mediterranean Intensive Agrosystems and Agri-Food Biotechnology (CIAMBITAL), University of Almería, Agri-Food Campus of International Excellence, ceiA3, 04120, Almería, Spain.

*Corresponding author: agarrido@ual.es

ORCID codes
Antonio Jesús Maldonado-Reina: 0000-0002-8457-6597
Rosalía López-Ruiz: 0000-0003-0806-9013
Roberto Romero-González: 0000-0002-2505-2056
José Luis Martínez Vidal: 0000-0003-0655-2597
Antonia Garrido-Frenich: 0000-0002-7904-7842

Table of contents

Table S1. Analysed difenoconazole and chlorantraniliprole-based commercial PPPs.

Table S2. Tentatively identified compounds in analysed PPPs.

Table S3. Toxicological information of identified co-formulants.

Table S1. Analysed difenoconazole and chlorantraniliprole-based commercial PPPs.

Commercial name	Formulation	Manufacturer	Composition (*w/v*)	Supplier
Altacor® 35 WG	WG	FMC International Switzerland	35 % Chlorantraniliprole **(*w/w*)**	Suministros Agrícolas Hnos. López S.A (La Mojonera, Almería)
Ampligo® 150 ZC	ZC	Syngenta Crop Protection AG	10 % Chlorantraniliprole 5 % Lambda-Cyhalothrin	Fito Agrícola S.L. (Castellón, Spain)
Ceremonia 25 EC®	EC	Globachem N.V.	25 % Difenoconazole	Agrares Iberia S.L. (Villanueva y Geltrú, Spain)
Cidely® Top	DC	Syngenta Crop Protection AG	12.5 % Difenoconazole	Suministros Agrícolas Hnos. López S.A (La Mojonera, Almería)
Coragen® 20 SC	SC	FMC International Switzerland	20 % Chlorantraniliprole	Sercopag Asesoría Agrícola (Bolanos de Calatrava, Spain)
Dagonis®	SC	BASF Agro B.V.	7.5 % Fluxapyroxad 5 % Difenoconazole	Suministros Agrícolas Hnos. López S.A (La Mojonera, Almería)
Duaxo®	EC	COMPO GmbH	1.67 % Difenoconazole	Leroy Merlin (Lezennes, France)
Dynali®	DC	Syngenta Crop Protection AG	6 % Difenoconazole 3 % Ciflufenamid	Nueva Pangea S.L. (Valdepeñas, Spain)
Kabuto® JED	EC	Kenogard	1.67 % Difenoconazole	Proag España S.L. (Villacarrillo, Spain)
Lexor-25	EC	Syngenta Crop Protection AG	25 % Difenoconazole	Jardinia Productos de Jardín S.L. (Elche, Spain)
Mavita® 250	EC	Adama	25 % Difenoconazole	Agro 21 S.L. (La Almunia de Doña Godina, Spain)
Nomada	EC	Globachem N.V.	25 % Difenoconazole	Fito Agrícola S.L. (Castellón, Spain)
Ortiva® Top	SC	Syngenta Crop Protection AG	20 % Azoxystrobin 12.5 % Difenoconazole	Suministros Agrícolas Hnos. López S.A (La Mojonera, Almería)
Score® 25 EC	EC	Syngenta Crop Protection AG	25 % Difenoconazole	Suministros Agrícolas Hnos. López S.A (La Mojonera, Almería)
Voliam® Targo	SC	Syngenta Crop Protection AG	4.5 % Chlorantraniliprole 1.8 % Abamectin	Suministros Agrícolas Hnos. López S.A (La Mojonera, Almería)

[a] Abbreviation: DC: dispersible concentrate; EC: emulsifiable concentrate; SC: suspension concentrate; WG: wettable granules; ZC: a mixture of capsule suspension (CS) in SC.

Table S2. Tentatively identified compounds in analysed PPPs.

Compound	Technique	Rt (min)	*m/z* Character	*m/z* Fragments	PPP	Total PPPs
Metilox	Unknown analysis	18.59	293.21112	291.19547 275.20056	P01	1 PPP (SC)
Dodecyl 4-hydroxybenzoate	Unknown analysis	18.91	307.22677	305.21112 289.21621	P01	1 PPP (SC)
PEG-13	Unknown analysis	11.25	564.35897	283.17513 221.13835	P01, P13, P15	3 PPP (SC, EC, SC)
PEG-11	Unknown analysis	10.50	503.3062		P01	1 PPP (SC)
PEG-14	Unknown analysis	11.83	635.38485	433.24321 265.16456	P01	1 PPP (SC)
1,2-Benzisothiazol-3(2H)-one	Unknown analysis	9.57	152.01646		P01, P12	2 PPPs (SC, ZC)
2-Butoxyethyl oleate	Unknown analysis	21.24	383.35197	362.27914 351.32576	P02, P14	2 PPPs (EC, EC)
PEG-4 sorbitan stearate	Unknown analysis	18.36	607.44157	563.41536 307.26316	P02, P14	2 PPPs (EC, EC)
PEG-3 dioleate	Unknown analysis	21.37	679.58712		P02, P14	2 PPPs (EC, EC)
4-sec-Butyl-2,6-di-tert-butylphenol	Unknown analysis	18.2	263.23694	245.22638 207.17434	P02, P14	2 PPPs (EC, EC)
Butyl linoleate	Unknown analysis	19.55	337.3101	281.24751 263.23694	P02, P14	2 PPPs (EC, EC)
Sodium 4-tetradecylbenzenesulfonate	Unknown analysis	18.81	353.21559		P02, P14	2 PPPs (EC, EC)
Sodium 4-tridecylbenzenesulfonate	Unknown analysis	18.38	339.19994		P02, P13, P14, P15	4 PPPs (EC, EC, EC, SC)
Sodium 4-dodecylbenzenesulfonate	Unknown analysis	18.06	325.18430		P02, P12, P13, P14, P15	5 PPPs (EC, ZC, EC, EC, SC)
Sodium 4-decylbenzenesulfonate	Unknown analysis	17.27	297.15299		P02, P03, P12, P14, P15	5 PPPs (EC, EC, ZC, EC, SC)
Sodium 4-nonylbenzenesulfonate	Unknown analysis	16.69	283.13734		P02, P14	2 PPPs (EC, EC)
Sodium 4-undecylbenzenesulfonate	Unknown analysis	17.35	311.16863		P03, P07, P08, P12, P13, P15	6 PPPs (EC, EC, EC, ZC, EC, SC)
Steareth-7	Unknown analysis	21.15	579.48305		P04, P08, P15	3 PPPs (EC, EC, SC)
Hexaethylene glycol monooctadecyl ether (Steareth-6)	Unknown analysis	21.15	535.45683		P04, P08	2 PPPs (EC, EC)
Ceteth-2 (Diethytlene glycol monohexadecyl ether)	Unknown analysis	20.69	331.32067		P04, P07, P08, P13	4 PPPs (EC, EC, EC, EC)

N,N-Diethyloctanamide	Unknown analysis	17.52	200.20089	198.18524 130.12264	P06	1 PPP (DC)
2-(4-Methyl-1-piperazinyl)ethanol	Unknown analysis	1.27	145.13354	143.11789 131.11789	P06	1 PPP (DC)
Mesityl phenyl ketone	Unknown analysis	17.79	225.12739	207.11683 195.11683	P07	1 PPP (EC)
Ethyl linoleate	Unknown analysis	19.78	307.26316	289.25259 239.20056	P07	1 PPP (EC)
Pentaethylene glycol monohexadecyl ether (C16E5) (Ceteth-5)	Unknown analysis	20.54	463.39932		P07	1 PPP (EC)
Hexaethylene glycol monohexadecyl ether (Ceteth-6)	Unknown analysis	20.54	507.42553		P07, P08, P13, P15	4 PPPs (EC, EC, EC, SC)
Heptaethylene glycol monohexadecyl ether (Ceteth-7)	Unknown analysis	20.5	551.45197		P07, P13	2 PPPs (EC, EC)
Octaethylene glycol monohexadecyl ether (Ceteth-8)	Unknown analysis	20.5	595.47796		P07	1 PPP (EC)
Nonaethylene glycol monohexadecyl ether (Ceteth-9)	Unknown analysis	20.5	639.50417		P07	1 PPP (EC)
Decaethylene glycol monohexadecyl ether (Ceteth-10)	Unknown analysis	20.5	683.53039		P07	1 PPP (EC)
Undecaethylene glycol monohexadecyl ether (Ceteth-11)	Unknown analysis	20.5	727.55660		P07	1 PPP (EC)
Triethytlene glycol monohexadecyl ether (Ceteth-3) (C16E3)	Unknown analysis	20.5	375.34689		P07, P08, P13	3 PPPs (EC, EC, EC)
Hexaethylene glycol monotetradecyl ether (Myreth-6)	Unknown analysis	19.9	479.39423		P07, P12	2 PPPs (EC, ZC)
4-Phenylcyclohexanone	Unknown analysis	15.88	175.11174	157.10118 145.10118	P07	1 PPP (EC)
Ceteth-4 (Tetraethytlene glycol monohexadecyl ether)	Unknown analysis	21.25	419.3731		P07, P08	2 PPP (EC, EC)
Castor oil diethanolamide	Unknown analysis	20.54	386.32649	331.28697 324.28971	P08	1 PPP (EC)
Tetraethylene glycol monooctadecyl ether (Steareth-4)	Unknown analysis	21.89	447.4044		P08, P13	2 PPPs (EC, EC)
Oleth-3	Unknown analysis	21.11	401.36254		P08, P13	2 PPPs (EC, EC)
PEG-9	Unknown analysis	10.62	415.25377	371.22756 177.11185	P08	1 PPP (EC)
Steareth-2 (Diethylene glycol monooctadecyl ether	Unknown analysis	22.07	359.35197		P08	1 PPP (EC)
Lauryldiemthylamine oxide	Unknown analysis	17.29	230.24784	133.10104 60.04439	P09	1 PPP (SC)
Trideceth-3	Unknown analysis	19.23	333.29994		P09, P10	2 PPPs (SC, SC)

Triethylene glycol monometyl ether (C1E3)	Unknown analysis	16.85	165.11214		P09	1 PPP (SC)
Hexaethylene glycol monodecyl ether (C10E6)	Unknown analysis	18.29	423.33163		P09	1 PPP (SC)
4-Phenylsulfonic acid	Unknown analysis	1.47	172.9914	93.03459<br>79.95736	P09	1 PPP (SC)
Monomethoxy PEG-11	Unknown analysis	11.71	517.32185	433.24321<br>177.11214	P10	1 PPP (SC)
Sodium 2,6-di-tert-butylnaphthalene-1-sulfonate	Unknown analysis	19.55	319.13734	79.95736	P11	1 PPP (WG)
D-Gluconic acid	Unknown analysis	1.50	195.05103	179.05611<br>165.04046	P11	1 PPP (WG)
D-Xylonic acid	Unknown analysis	1.59	165.04046	147.0299<br>129.01933	P11	1 PPP (WG)
Stearyldiethanolamine	Unknown analysis	21.29	358.36796	340.35739<br>314.34174	P11	1 PPP (WG)
Sodium 1-butyl-1-naphthalenesulfonate	Unknown analysis	16.87	263.07474		P11	1 PPP (WG)
Sodium 2-butyl-1-naphthalenesulfonate	Unknown analysis	16.87	263.07474		P11	1 PPP (WG)
Sodium 8-(2-methyl-2-undecanyl)-1-naphthalenesulfonate	Unknown analysis	20.78	375.19994		P11	1 PPP (WG)
Sodium 1-naphthalenesulfonate	Unknown analysis	7.7	207.01214		P11	1 PPP (WG)
Sodium 2-naphthalenesulfonate	Unknown analysis	8.99	207.01214		P11	1 PPP (WG)
N,N-Bis(2-hydroxyethyl)dodecanamide (Lauramide DEA)	Unknown analysis	20.01	288.25332	226.21654<br>106.08626	P11	1 PPP (WG)
Sodium decyl sulfate	Unknown analysis	19.2	237.1166	96.9589	P11	1 PPP (WG)
1-Dodecylnaphthalene	Unknown analysis	23.6	255.21073	129.06988	P11	1 PPP (WG)
2-Dodecylnaphthalene	Unknown analysis	23.6	255.21073	129.06988	P11	1 PPP (WG)
1-Nonylnaphthalene	Unknown analysis	21.16	255.21073		P11	1 PPP (WG)
1-Hexadecylnaphthalene	Unknown analysis	24.35	353.32028	185.13248<br>129.06988	P11	1 PPP (WG)
2-Hexadecylnaphthalene	Unknown analysis	24.57	353.32028	185.13248<br>129.06988	P11	1 PPP (WG)
1-Monopalmitin	Unknown analysis	20.35	331.28429	125.09609<br>177.11214	P12	1 PPP (ZC)
Triethylene glycol monotetradecyl ether (Myreth-3) (C14E3)	Unknown analysis	19.93	345.30103		P13	1 PPP (EC)
Oleth-4	Unknown analysis	21.11	401.36254		P15	1 PPP (SC)
Tetrathylene glycol monotetradecyl ether (Myreth-4) (C14E4)	Unknown analysis				P15	1 PPP (SC)
2-Palmitoylglicerol	Suspect screening	19.79	331.28429	313.27372<br>257.24751	All except	13 PPPs

					for P06, P11	
9-Octadecenamide	Suspect screening	19.70	282.27914		P02, P14	2 PPPs (EC, EC)
Glyceryl monostearate	Suspect screening	20.45	359.31559		All except for P06, P11	13 PPPs
2-[2-[4-(1,1,3,3-tetramethylbutyl)phenoxy]ethoxy]ethano	Suspect screening	17.95	295.22589		P02, P14	2 PPPs (EC, EC)
17-(4-Nonylphenoxy)-3,6,9,12,15-pentaoxaheptadecan-1-ol	Suspect screening	20.28	485.34728		P04, P07, P08	3 PPPs (EC, EC, EC)
20-(4-Nonylphenoxy)-3,6,9,12,15,18-hexaoxaicosan-1-ol	Suspect screening	20.32	529.37350		P07, P08	2 PPPs (EC, EC)
Aniline	Suspect screening	1.41	94.06513		P01,P10, P11,P12	4 PPPs (SC, SC, WG, ZC)
Dipropylene glycol methyl ether	Suspect screening	7.01	149.11722	95.04914 89.05971	P02, P14	2 PPPs (EC, EC)
Methylchloroisothiazolinone	Suspect screening	7.69	149.97749		P10	1 PPP (SC)
N,N-dimethyldecanamide	Suspect screening	17.52	200.20089		P03, P05, P06	3 PPPs (EC, DC, DC)
1-methylpyrrolidin-2-one	Suspect screening	2.35	100.07569	58.02874	P04, P05, P06, P07, P08, P15	6 PPPs (EC, DC, DC, EC, EC, SC)
Nonaethylene glycol monododecyl ether	Suspect screening	18.74	583.44157		P03, P04, P07, P08, P10	5 PPPs (EC, EC, EC, EC, SC)

Abbreviation: P01: Voliam Targo; P02: Kabuto JED; P03: Ceremonia; P04: Mavita; P05: Cidely Top; P06: Dynali; P07: Lexor; P08: Score 25; P09: Dagonis; P10: Coragen 20 SC; P11: Altacor; P12: Ampligo; P13: Nomada; P14: Duaxo; P15: Ortiva Top

Table S3. Toxicological information of identified co-formulants.

Co-formulant	Oral RfD (mg/kg/day)	RfC (mg/m^3)	NOAEL (mg/kg/day)	LD_{50} (g/kg)	Reference
1,2-Benzisothiazol-3(2H)-one	0.017	NA	5	NA	**1**
Aniline	0.007	0.001	3.4 mg/m^3	NA	**2, 3**
Alkylnaphthalene sulfonates	0.5	NA	50	NA	**4**
Sodium dodecyl sulfate (SDS)	1	NA	100	NA	**5**
Alkylbenzene sulfonates	0.5	NA	50	NA	**6**
Ceteth-2 (Alkyl ethoxylate)	NA	NA	NA	> 25.1	**7**
Ceteth-10 (Alkyl ethoxylate)	NA	NA	NA	2.5	**7**
Ceteth-20 (Alkyl ethoxylate)	NA	NA	NA	3.59	**7**

[a]Abbreviation: LD_{50}: Median lethal dose; NA: Not available; NOAEL: Non-observed-adverse-effect level; RfC: Reference concentration; RfD: Reference dose.

[1]https://archive.epa.gov/pesticides/reregistration/web/pdf/benzisothiazolin_red.pdf

[2]https://iris.epa.gov/ChemicalLanding/&substance_nmbr=350

[3]https://www.michigan.gov/documents/deq/deq-rrd-chemAnilineDatasheet_527727_7.pdf

[4]https://www.govinfo.gov/content/pkg/FR-2009-08-05/pdf/E9-18702.pdf

[5]https://www.govinfo.gov/content/pkg/FR-2009-08-12/pdf/E9-19314.pdf

[6] https://archive.epa.gov/pesticides/reregistration/web/pdf/alkylbenzene_red.pdf

[7] https://journals.sagepub.com/doi/10.1177/109158189901800203
